# Association of Interstitial Cystitis/Bladder Pain Syndrome with Stress-Related Diseases: A Nationwide Population-Based Study

**DOI:** 10.3390/jcm10235669

**Published:** 2021-11-30

**Authors:** Min-Hsin Yang, Jing-Yang Huang, Sung-Lang Chen, James Cheng-Chung Wei

**Affiliations:** 1 Institute of Medicine, Chung Shan Medical University, Taichung 402, Taiwan; barbarian0607@icloud.com (M.-H.Y.); wchinyang@gmail.com (J.-Y.H.); 2 Department of Urology, Chung Shan Medical University Hospital, Taichung 402, Taiwan; urologychen@gmail.com; 3 Center for Health Data Science, Chung Shan Medical University Hospital, Taichung 402, Taiwan; 4 School of Medicine, Chung Shan Medical University, Taichung 402, Taiwan; 5 Graduate Institute of Integrated Medicine, China Medical University, Taichung 402, Taiwan; 6 Department of Allergy, Immunology and Rheumatology, Chung Shan Medical University Hospital, Taichung 402, Taiwan

**Keywords:** stress, stress-related disease, interstitial cystitis/bladder pain syndrome, national-based study

## Abstract

Background: Stress-related diseases (SRDs) are adjustment disorders triggered by stressful life changes. There is a growing body of evidence showing that stress plays an important role in the pathophysiology of IC/BPS. In the present study, we investigated the association between SRDs and a subsequent association of interstitial cystitis/bladder pain syndrome (IC/BPS). Methods: We performed a nested case-control study from the Longitudinal Health Insurance Database (LHID) of Taiwan. The two-year time-varying association between SRDs and IC/BPS was explored to distinguish the short- or long-term effects of these factors. We then conducted multiple conditional logistic regressions to evaluate the adjusted odds ratio (OR) of IC/BPS in patients with a history of SRDs. Results: A total of 1103 IC/BPS patients and 4412 non-IC/BPS patients were analyzed. For all SRDs, the significantly increased risks were obtained in 2 years before IC/BPS diagnosis, and the higher OR was observed within 3 months before the diagnosis of IC/BPS. Multiple conditional logistic regressions showed that patients who had prior medical care for urinary tract infection (OR = 10.95, 95% CI = 9.07 to 13.22), chronic obstructive pulmonary disease (OR = 1.48, 95% CI = 1.13 to 1.93), peptic ulcer (OR = 1.69, 95% CI = 1.37 to 2.09), inflammatory bowel syndrome (OR = 1.66, 95% CI = 1.21 to 2.29), autoimmune diseases (OR = 1.48, 95% CI = 1.11 to 1.97), depression (OR = 1.54, 95% CI = 1.24 to 1.91), sleep disorders (OR = 1.45, 95% CI = 1.19 to 1.78), and allergic rhinitis (OR = 1.29, 95% CI = 1.03 to 1.62) within 2 years had a significant risk of IC/BPS. Conclusions: Our study demonstrates that the health care for SRDs within the previous 2 years is associated with an increased risk of subsequent IC/BPS. The time-varying association provides an important insight that helps us to identify cases with IC/BPS, especially among patients with repeated UTI visits.

## 1. Introduction

Interstitial cystitis/bladder pain syndrome (IC/BPS) is a common disease with a prevalence ranging from 2.7~6.5%, which represents 3.3 to 7.9 million women 18 years or older in the United States [1]. Common symptoms of IC/BPS include painful urination, increased frequency and/or urgency, pelvic tenderness, and extragenital discomfort in the back or lower abdomen [2]. Diagnosis is based mainly on the exclusion of other bladder diseases, such as urinary tract infection, overactive bladder, and gynecological or colorectal diseases [2]. Treatment includes lifestyle modifications, oral or intravesical pharmacological therapy, neuromodulation, or surgical intervention [3].

Although IC/BPS is very common, it can face serious challenges due to delayed diagnosis and difficult management [4,5]. Previous research has reported that only 9.7% of women with bladder pain symptoms are diagnosed with IC/BPS [1]. The main reason is that the etiology of IC/BPS is heterogeneous and not fully elucidated. There are many possible pathologies mentioned in the literature, including urothelial dysfunction, mast cell activation, antiproliferative factor, genetics, autoimmunity, and central sensitization [6]. The lack of evidence and the various definitions make it difficult to establish a correct algorithm for the diagnosis of IC/BPS [7]. Patients often struggle for a long time before treatment for lack of a proper diagnosis, affecting their mental health and work [7]; they are six times more likely to lose work time [8]. Early diagnoses of IC/BPS could improve both global and symptom-specific quality of life, knowing the preceding disease can facilitate early diagnosis [9,10].

Stress-related disorders refer to adjustment disorders triggered by identifiable and stressful life changes [11]. They are known to induce many stress-related diseases (SRDs), such as autoimmune disease, depression, peptic ulcer, and asthma, among others [12,13,14]. There is a growing body of evidence showing that stress plays an important role in the pathophysiology of IC/BPS. Some studies have reported the overexpression of stress-related hormones and receptors in the bladder of IC/BPS patients [15,16,17]. Previous observational studies have reported that many SRDs may coexist with IC/BPS, including depression, anxiety, asthma, IBD, autoimmune disease, and so forth [18]. However, no study has reported the association between SRDs and IC/BPS. In the present study, we investigated the timeline correlation between SRDs and subsequent diagnosis with IC/BPS using Taiwan’s nationwide database. The results of this study can help to better understand the potential etiology and improve early diagnosis of IC/BPS.

## 2. Materials and Methods

We performed a nested case-control study from the archives of the Longitudinal Health Insurance Database (LHID) 2000. This study was approved by the institutional review board of the Chung Shan Medical University Hospital (IRB CS15134). In accordance with Taiwan’s computer-processed personal data protection law, all identities of individuals or institutions were encrypted before analysis; therefore, the need for informed consent was waived.

- Data source

**Figure 1:**
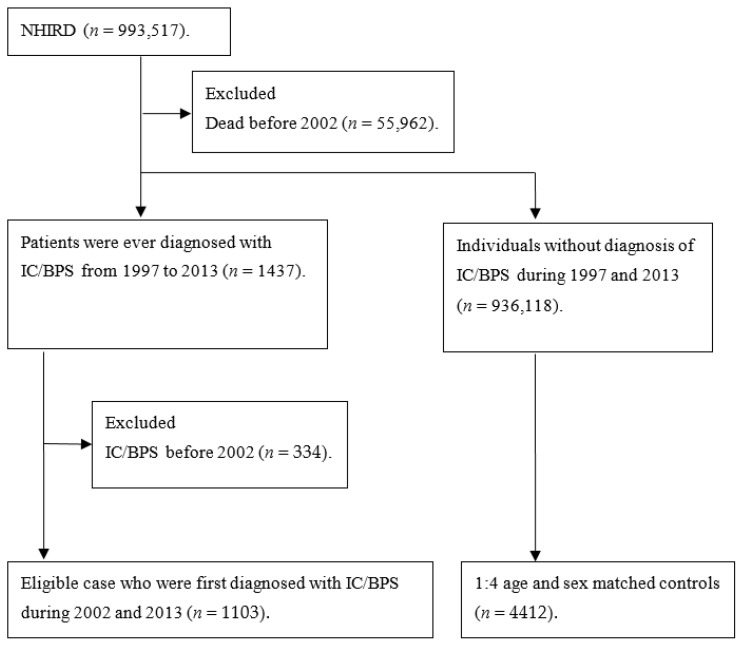
Flow chart of selection of study population in the nested case-control design: NHIRD, National Health Insurance Research Datasets; IC/BPS, interstitial cystitis/bladder pain syndrome.

The LHID 2000 contains insurance claim datasets, including registration files and original claim data for reimbursement, which are the subsets of the National Health Insurance Research Datasets (NHIRD). NHIRD is derived from the National Health Insurance (NHI) program that covered about 93% of residents in 1996 and 99% in 2011 under the National Health Insurance Administration, Ministry of Health and Welfare, Taiwan. The National Health Research Institutes conducted the NHIRD, and the LHID 2000 comprised one million individuals who were insured from 1996 to 2000, randomly sampled from the NHIRD. The LHID provides information about expenditure, diagnosis, prescription of drugs, and medical orders in ambulatory and inpatient care. In this study, the time frame of LHID 2000 was between 1997 and 2013. However, the LHID 2000 was truncated, and the records of diagnoses were not ICD-9-CM coded before 2000; therefore, we excluded cases that were deceased before 2002 to ensure that all cases could be observed for at least 2 years before the index date (Figure 1).

- Cases of IC/BPS

The flow chart of the selection of the study population is shown in Figure 1. We identified patients who were newly diagnosed with IC/BPS from 1 January 2002 through to 31 December 2013 by ICD-9 code 595.1. For each IC/BPS case, we defined the first date of diagnosis of IC/BPS as the index date, and the related factors were observed within 2 years before the index date. The diagnosis of IC/BPS was defined as at least three outpatient visits or one diagnosis during hospitalization for IC/BPS. Furthermore, to improve the diagnosis validity, all IC/BPS subjects included in this study received a prescription for Cystistat (hyaluronic acid). As described in previous studies, Cystistat is tightly regulated in the NHI program [19]. By regulation of the NHI, prescriptions of Cystistat must be reviewed in the patient’s medical history. Therefore, it improved the accuracy of diagnosis of IC/BPS and those who would be eligible for this medication, decreasing the misclassification of cases in our study.

- Control of non-IC/BPS

We selected four non-IC/BPS controls who were individually age- (the difference was less than 1 year) and sex-matched with one IC/BPS case at the index date. We applied the algorithm to allocate the case’s index date for controls. If the selected controls died or withdrew from the NHI program, we resampled an alternative control until four appropriate controls were selected. Among the enrolled cases and controls, we identified the related factors, including demographics and diagnosis of disease, within 2 years before the index date.

- History of comorbidity and SRDs

We selected the comorbidities, including hyperlipidemia (ICD-9 code 272), diabetes mellitus (code 250), hypertension (codes 401–405), coronary artery disease (codes 410–414), stroke (codes 430–438), chronic kidney disease (code 585), chronic liver diseases (codes 571 and 573), and gout (code 274). SRDs were selected in accordance with previous studies and included chronic obstructive pulmonary disease (COPD) (ICD-9 codes 490–492 and 493–496), asthma (code 493), allergic rhinitis (code 477), atopic dermatitis (code 691), peptic ulcer disease (codes 531–534), inflammatory bowel syndrome (IBS) (codes 555–556), autoimmune disease (code—see Supplementary Table S1), depression (codes 296, 300, 309, and 311), and sleep disorders (code—see Supplementary Table S2). Those factors were identified within 2 years before the index date (excluding the index date). Other covariates—sex, age, urbanization, and length of hospital stays at baseline—were also considered in this study. Urinary tract infection (UTI) (ICD-9 codes 599.0, 595.0, and 595.9) was also included for analysis. Because IC/BPS was diagnosed mainly based on exclusion, many patients tended to be diagnosed as having a UTI during the diagnostic process.

- Statistical analysis

Demographic characteristics, length of hospital stay, history of disease diagnosis, and medications are presented as frequencies and proportions. A conditional logistic regression was used to evaluate the odds ratio (95% confidence interval) of IC/BPS for univariate analysis. To understand the time-varying association between SRDs and IC/BPS, we explored the odds ratio in different time periods before the diagnosis of IC/BPS (within 3 months, 3–6 months, 6 months to 1 year, and 1–2 years) to distinguish the short- or long-term effects of these factors (Figure S1).

We conducted three multiple conditional logistic regression to compare the model fit when predicting the probability of IC/BPS. The first model contained demographic variables. The second model added SRDs that appeared within 2 years before the index date. The third model replaced the SRDs that appeared within 3 months before the index date in the second model. All statistical tests were two-sided, and *p*-values of <0.05 were considered statistically significant. All data and statistics were processed and analyzed using SAS statistical software version 9.4 (SAS Institute, Cary, NC, USA).

## 3. Results

A total of 1103 IC/BPS patients and 4412 non-IC/BPS controls were analyzed. Females predominated (81.5%), and the age distribution was mostly 35–65 years old. Table 1 shows that the case group was distributed more in urban areas than the control group. The comorbidities were identified within 2 years before IC/BPS; cases had a higher prevalence than controls for hyperlipidemia, hypertension, coronary artery disease, stroke, chronic kidney disease, and chronic liver disease. With regard to SRDs, cases had a higher prevalence of COPD, peptic ulcer, IBS, autoimmune disease, depression, sleep disorder, asthma, and allergic rhinitis.

Table 2 shows the potential risk factors of IC/BPS according to the different time periods before diagnosis of IC/BPS. Some SRDs (COPD, peptic ulcer, sleep disorders, and allergic rhinitis) were risk factors across every period within 2 years. Some SRDs appeared to be a risk factor when closer to the diagnosis of IC/BPS (within 1 year: autoimmune diseases, depression; within 6 months: atopic dermatitis). All SRDs appeared to be risk factors within 3 months before the diagnosis of IC/BPS. UTI also showed a strong timeline correlation before diagnosis of IC/BPS across all time periods. The significant risk of UTI rise appeared when closer to the diagnosis of IC/BPS (6 months: OR 13.1; 3 months: OR 21.4). Patients who had long-term seeking medical care for UTI within 2 years had a higher risk of IC/BPS (only visit within 3 months: OR 20.1; visit during 3–6 months and within 3 months before index date: OR 45.3; visit during 6–12 months, 3–6 months, and within 3 months before index date: OR 74.2; visit during 12–24 months, 6–12 months, 3–6 months, and within 3 months before index date: OR 128.6) (Table S3). To demonstrate which ADs contribute as risk factor, the ADs were analyzed separately in Table S4. Except for inflammatory bowel diseases (Crohn’s disease, ulcerative colitis), only Sjogren syndrome was risk factor for IC/BPS in autoimmune diseases.

Table 3 shows the adjusted ORs comprising baseline characteristics (urbanization, comorbidity) and potential risk of medical disease. Model 2 included all covariates within 2 years before the index date. The results showed COPD, peptic ulcer, IBS, autoimmune diseases, depression, sleep disorders, and allergic rhinitis had a significant OR for IC/BPS. Model 3 replaced the morbidities within 3 months before the index date in Model 2. The results showed peptic ulcer, IBS, depression, sleep disorders, and allergic rhinitis had a significant OR for IC/BPS, and these SRDs demonstrated a higher OR when they appeared closer to IC/BPS. We further evaluated the association between repeat diagnosis in the different time periods of SRDs, UTI, and the change of ORs in Table S3. The result revealed that UTI and some SRDs (peptic ulcer, depression, sleep disorders) diagnosed within 3 months accounted for the most important role for IC/BPS.

## 4. Discussion

To our knowledge, this is the first study to address SRDs and their time-varying associations with the onset of IC/BPS. The time-varying analysis showed that most SRDs (COPD, peptic ulcer, IBS, autoimmune diseases, depression, sleep disorders, allergic rhinitis) were a risk factor for IC/BPS when the diagnosis had been made within 2 years. Regarding the SRDs that were diagnosed within 3 months, peptic ulcer, IBS, depression, sleep disorders, and allergic rhinitis had a higher risk for subsequent IC/BPS. Furthermore, we also noted that the patients with a recent and repeat diagnosis of UTI were strongly associated with IC/BPS.

Stress is believed to alter an array of physiologic factors, including disruption of the autonomic nervous system, the hypothalamic-pituitary-adrenal axis, and multiple bodily systems (e.g., immune function), thus exacerbating susceptibility to stress-related diseases (SRDs). Previous animal studies in the literature have indicated an association between stress and IC/BPS. For example, some have revealed that chronic psychological stress can trigger visceral hyperalgesia, increased expression of the nerve growth factor, and increased numbers of mast cells in the bladder mucosa, thereby increasing voiding frequency [16,20,21]. Chronic stress exposure early in life has been shown to increase the likelihood of pelvic pain later in life [17]. Several epidemiological studies have also revealed the association between SRDs and IC/BPS. Alagiri et al. conducted a large-scale survey that included 2406 patients diagnosed with IC/BPS. The study reported that inflammatory bowel disease, systemic lupus erythematosus, allergies, IBS, sensitive skin, and fibromyalgia all have an increased association with IC/BPS [22]. Big data analyses using the Taiwan Health Insurance Database have also confirmed that the prevalence of these diseases increases in patients with IC/BPS [19]. In addition, many other SRDs were reported to be associated with IC/BPS, including anxiety, depression, reflux esophagitis, peptic ulcer, rheumatoid arthritis, Sjogren syndrome, and COPD [23,24,25,26,27,28]. In our analysis, most SRDs more frequently appeared within 2 years of the diagnosis of IC/BPS; surprisingly, all SRDs more frequently appeared within 3 months. In multivariate analysis, many SRDs were a risk factor for IC/BPS within 2 years. Regarding the short-term period, peptic ulcer, IBS, depression, sleep disorders, and allergic rhinitis had higher risk factors for subsequent IC/BPS within 3 months. However, we noted that the risk of some SRDs became stronger when the diagnosis of SRDs was made closer to the onset of IC/BPS, as shown in Table S3. The current study still could not support the causal relationship between SRDs and IC/BPS; further research is necessary.

In this study, we noted that 70% of the patients with IC/BPS received a diagnosis of UTI before the index day (the day IC/BPS was first diagnosed). In our opinion, the high prevalence of UTI before IC/BPS represents a diagnostic process that excludes other etiology. As mentioned above, diagnosing IC/BPS is difficult and time-consuming. It is reasonable for a physician to diagnose and treat the condition as UTI initially, especially when the symptoms of IC/BPS are multifocal and not well established. Although the identification of UTI might not be accurate in this study, as it was based merely on a diagnostic code in the database, the finding of a high prevalence of UTI is still meaningful. Based on our findings, patients diagnosed with UTI in the past 2 years were associated with future IC/BPS. The risk rose significantly when the diagnosis of UTI was repeatedly made recently (Table S3). It is reasonable to suspect IC/BPS when a patient has been diagnosed with UTI repeatedly, has responded poorly to antibiotic treatment, or does not match the clinical scenario.

In our study, patients with IC/BPS were more prevalent in urban cities and was a risk factor for IC/BPS in the multivariate analysis. A similar result was also noted by a previous study that reported region variability and a higher prevalence in higher-income populations [25]. There were several possible reasons. First, people who live in urban cities exposed to a higher stress environment, which could induce IC/BPS. Second, people who live in rural cities lack medical resources (fewer medical facilities, fewer urologists), and they are prone to delay the diagnosis of diseases.

According to the results of our study, patients who reported being repeatedly diagnosed with unusual UTI in combination with SRDs during the past 2 years could be considered a reference for the diagnosis of IC/BPS. However, in cases of misdiagnosis and delayed treatment of another etiology, a rigorous diagnosis process should still be followed, as indicated in current guidelines [29].

The strength of our study is the use of nationwide population-based data to establish a regression model of IC/BPS. Advantages of using our NHIRD in research include an enormous sample size, population-based data, and long-term comprehensive follow-up [30]. Furthermore, the use of a nationwide population-based database in our study resulted in stronger validity of the model. In addition, we experimented with different models to clarify the best-fitted model. Nevertheless, there are several limitations related to the LHID 2000 database. First, the ICD codes for diagnoses of IC/BPS and SRDs were based on administrative claims data recorded by physicians and hospitals rather than in a prospective clinical setting. Despite the fact that we strictly defined disease as three outpatient clinic diagnostic codes or one inpatient diagnostic code, misclassification may still exist. However, we included IC/BPS patients who were prescribed sodium hyaluronate (Cystistat), which requires a strict pre-review based on Taiwan NHI regulations [31]. The misclassification of IC/BPS could be minimized. Second, due to limited availability in the LHID 2000, we could not assess all underlying medical comorbidities. Additionally, some SRDs that have been reported as being associated with IC/BPS such as pelvic inflammatory disease could not be identified in this study [32]. Third, the database was limited in adjusting for other possible confounding factors due to the lack of information about family history, lifestyle, dietary habits, alcohol consumption, cigarette smoking, or body mass index. Fourth, we conducted an age- and sex-matched case-control study design, which limited the estimation of the effect of age and sex on the diagnosis of IC/BPS; however, we examined the relationship between SRDs with IC/BPS when we adjusted for the confounding effects of age and sex.

## 5. Conclusions

In conclusion, our study demonstrates that most SRDs are associated with an increased risk of subsequent IC/BPS, especially when peptic ulcer, IBS, depression, sleep disorders, and allergic rhinitis appeared in the past 3 months. Clinicians should suspect this differential diagnosis when symptoms of bladder discomfort that are repeatedly diagnosed as urinary tract infection accompany a recent history of an SRD. Although these indications cannot serve as criteria for a definitive diagnosis of IC/BPS, being aware of their correlations could improve the early diagnosis of IC/BPS.

## Figures and Tables

**Table 1:** Baseline characteristics among study groups.

Variables	Control	IC/BPS	Univariate OR (95% CI)	*p*
*n*	4412	1103		
Sex				
Female	3596 (81.5%)	899 (81.5%)		
Male	816 (18.5%)	204 (18.5%)		
Age (matched ± 1-year-old)				
<35	989 (22.4%)	249 (22.57%)		
35–50	1355 (30.7%)	334 (30.28%)		
50–65	1135 (25.7%)	286 (25.93%)		
>=65	933 (21.2%)	234 (21.21%)		
Residence area				
Taipei city (capital city)	1754 (39.8%)	553 (50.14%)	Reference	
North district	577 (13.1%)	133 (12.06%)	0.74 (0.60 to 0.91)	0.005
Central district	773 (17.5%)	120 (10.88%)	0.49 (0.40 to 0.61)	<0.001
South district	577 (13.1%)	167 (15.14%)	0.92 (0.75 to 1.12)	0.397
Kaohsiung city	653 (14.8%)	112 (10.15%)	0.54 (0.43 to 0.67)	<0.001
East district	78 (1.8%)	18 (1.63%)	0.75 (0.45 to 1.27)	0.284
Urbanization				
Urban	2688 (60.9%)	721 (65.37%)	Reference	
Sub-urban	1268 (28.7%)	295 (26.75%)	0.87 (0.75 to 1.01)	0.063
Rural	456 (10.3%)	87 (7.89%)	0.71 (0.55 to 0.91)	0.006
Length of hospitalized stays, days				
0	3668 (83.1%)	815 (73.9%)	Reference	
1–6	418 (9.5%)	149 (13.5%)	1.63 (1.33 to 2.00)	<0.001
>=7	326 (7.4%)	139 (12.6%)	2.02 (1.62 to 2.53)	<0.001
Comorbidities (ever diagnosed within 2 years before index date)				
UTI	747 (16.9%)	771 (69.9%)	12.38 (10.40 to 14.73)	<0.001
Hyperlipidemia	652 (14.8%)	234 (21.2%)	1.67 (1.40 to 2.01)	<0.001
Diabetes mellitus	506 (11.5%)	157 (14.2%)	1.33 (1.08 to 1.63)	0.007
Hypertension	1006 (22.8%)	298 (27.0%)	1.39 (1.16 to 1.67)	<0.001
Coronary artery disease	403 (9.1%)	138 (12.5%)	1.51 (1.21 to 1.89)	<0.001
Stroke	217 (4.9%)	75 (6.8%)	1.49 (1.11 to 2.01)	0.008
Chronic kidney disease	55 (1.3%)	26 (2.4%)	1.93 (1.20 to 3.10)	0.007
Chronic liver diseases	430 (9.8%)	193 (17.5%)	2.00 (1.66 to 2.41)	<0.001
Gout	219 (5.0%)	76 (6.9%)	1.44 (1.09 to 1.89)	0.010
COPD	395 (9.0%)	185 (16.8%)	2.21 (1.81 to 2.71)	<0.001
Peptic ulcer	619 (14.0%)	364 (33.0%)	3.11 (2.66 to 3.64)	<0.001
IBS	161 (3.7%)	143 (13.0%)	3.99 (3.13 to 5.07)	<0.001
Autoimmune disease	318 (7.2%)	128 (11.6%)	1.69 (1.36 to 2.11)	<0.001
Depression	622 (14.1%)	355 (32.2%)	2.93 (2.51 to 3.42)	<0.001
Sleep disorders	830 (18.8%)	401 (36.4%)	2.56 (2.21 to 2.98)	<0.001
Asthma	231 (5.2%)	96 (8.7%)	1.75 (1.36 to 2.25)	<0.001
Allergic rhinitis	555 (12.6%)	228 (20.7%)	1.81 (1.53 to 2.15)	<0.001
Atopic dermatitis	122 (2.8%)	41 (3.7%)	1.36 (0.95 to 1.96)	0.094

IC/BPS, interstitial cystitis/bladder pain syndrome; UTI, urinary tract infection; COPD, chronic obstructive pulmonary disease; IBS, irritable bowel syndrome.

**Table 2:** The odds ratio of interstitial cystitis/bladder pain syndrome by time-varying defined variable.

	Odds Ratio (95% CI) by Different Time Windows before Index Date			
	−24 to −12 Months	−12 to −6 Months	−6 to −3 Months	−3 Months to Index
Comorbidities				
UTI	6.45 (5.46 to 7.63)	8.27 (6.89 to 9.93)	13.11 (10.29 to 16.69)	21.39 (17.05 to 26.84)
COPD	1.90 (1.49 to 2.43)	2.12 (1.59 to 2.81)	1.77 (1.23 to 2.54)	2.19 (1.54 to 3.12)
Peptic ulcer	2.72 (2.26 to 3.26)	3.38 (2.74 to 4.16)	2.68 (2.09 to 3.42)	3.48 (2.73 to 4.42)
IBS	1.60 (0.87 to 2.92)	3.25 (1.56 to 6.76)	2.86 (0.91 to 9.00)	4.00 (1.67 to 9.61)
Autoimmune disease	1.27 (0.95 to 1.68)	1.96 (1.44 to 2.66)	1.59 (1.12 to 2.27)	1.75 (1.22 to 2.49)
Depression	2.39 (2.00 to 2.85)	3.15 (2.58 to 3.83)	2.71 (2.18 to 3.37)	3.79 (3.06 to 4.69)
Sleep disorders	2.32 (1.96 to 2.75)	2.39 (1.98 to 2.89)	2.12 (1.71 to 2.61)	2.63 (2.13 to 3.24)
Asthma	1.57 (1.15 to 2.16)	1.45 (0.98 to 2.14)	1.52 (0.96 to 2.41)	2.08 (1.30 to 3.32)
Allergic rhinitis	1.68 (1.36 to 2.07)	1.51 (1.16 to 1.95)	1.92 (1.42 to 2.61)	1.95 (1.42 to 2.68)
Atopic dermatitis	1.62 (1.03 to 2.55)	1.13 (0.61 to 2.10)	3.20 (1.26 to 8.11)	2.26 (1.15 to 4.46)

IC/BPS, interstitial cystitis/bladder pain syndrome; UTI, urinary tract infection; COPD, chronic obstructive pulmonary disease; IBS, irritable bowel syndrome.

**Table 3:** Conditional logistic regression for estimating odds ratio of interstitial cystitis in specific variables.

Variables	Model 1	Model 2	Model 3
Urbanization			
Urban	Reference	Reference	Reference
Sub-urban	1.06 (0.90 to 1.26)	1.23 (0.99 to 1.51)	1.19 (0.96 to 1.47)
Rural	0.80 (0.61 to 1.03)	0.80 (0.58 to 1.11)	0.82 (0.59 to 1.15)
Residence area			
Taipei city (capital city)	Reference	Reference	Reference
North district	0.71 (0.57 to 0.88)	0.73 (0.55 to 0.95)	0.77 (0.59 to 1.00)
Central district	0.48 (0.38 to 0.61)	0.36 (0.27 to 0.48)	0.35 (0.26 to 0.48)
South district	0.93 (0.75 to 1.15)	0.89 (0.68 to 1.17)	0.84 (0.64 to 1.11)
Kaohsiung city	0.54 (0.43 to 0.68)	0.44 (0.33 to 0.58)	0.44 (0.33 to 0.59)
East district	0.76 (0.45 to 1.29)	0.72 (0.38 to 1.37)	0.46 (0.22 to 0.94)
Length of hospitalized stays, days			
0	Reference	Reference	Reference
1–6	1.68 (1.36 to 2.06)	1.12 (0.86 to 1.46)	1.20 (0.91 to 1.58)
>=7	2.05 (1.63 to 2.56)	1.01 (0.75 to 1.37)	1.56 (1.16 to 2.12)
Comorbidities		[Within 2 years]	[Within 3 months]
UTI		10.95 (9.07 to 13.22)	20.96 (16.44 to 26.71)
Hyperlipidemia		1.29 (1.00 to 1.66)	1.13 (0.79 to 1.62)
Diabetes mellitus		0.95 (0.72 to 1.25)	0.95 (0.68 to 1.33)
Hypertension		1.05 (0.82 to 1.34)	1.00 (0.76 to 1.31)
Coronary artery disease		0.80 (0.60 to 1.08)	0.94 (0.61 to 1.45)
Stroke		1.08 (0.74 to 1.57)	1.22 (0.72 to 2.09)
Chronic kidney disease		1.15 (0.62 to 2.13)	0.77 (0.30 to 1.94)
Chronic liver diseases		1.41 (1.09 to 1.81)	1.27 (0.82 to 1.97)
Gout		1.01 (0.70 to 1.44)	0.62 (0.33 to 1.18)
Stress-related diseases			
COPD		1.48 (1.13 to 1.93)	1.57 (0.99 to 2.50)
Peptic ulcer		1.69 (1.37 to 2.09)	2.11 (1.52 to 2.93)
IBS		1.66 (1.21 to 2.29)	2.36 (1.26 to 4.39)
Autoimmune disease		1.48 (1.11 to 1.97)	1.54 (0.95 to 2.48)
Depression		1.54 (1.24 to 1.91)	2.04 (1.52 to 2.75)
Sleep disorders		1.45 (1.19 to 1.78)	1.59 (1.18 to 2.15)
Asthma		0.90 (0.64 to 1.26)	1.31 (0.68 to 2.51)
Allergic rhinitis		1.29 (1.03 to 1.62)	1.70 (1.12 to 2.59)
Atopic dermatitis		0.97 (0.60 to 1.56)	2.47 (0.96 to 6.34)

IC/BPS, interstitial cystitis/bladder pain syndrome; UTI, urinary tract infection; COPD, chronic obstructive pulmonary disease; IBS, irritable bowel syndrome.

## Data Availability

The original data were limited according to the National Health Insurance policy, which was not available outside the facility.

## Supplementary Materials

The following are available online at https://www.mdpi.com/article/10.3390/jcm10235669/s1, Figure S1: Time period of the stress related disease before the diagnosis of IC/BPS (index date), Table S1: ICD-9 diagnostic codes of 33 autoimmune diseases, Table S2: ICD-9 diagnostic codes of sleep disorders, Table S3: The repeat diagnosis of SRDs and odd ratio of the diagnosis of IC/BPS, Table S4: Morbidities of autoimmune disease among study groups.
