# Supplementary material for: Association of Interstitial Cystitis/Bladder Pain Syndrome with Stress-Related Diseases: A Nationwide Population-Based Study
Source: J Clin Med. 2021 Nov 30;10(23):5669. doi: 10.3390/jcm10235669 (PMC8658298; doi:10.3390/jcm10235669)
Supplement: Supplementary file 1 [file jcm-10-05669-s001.zip › jcm-1439900-supplementary.pdf]

## Supplementary Materials:

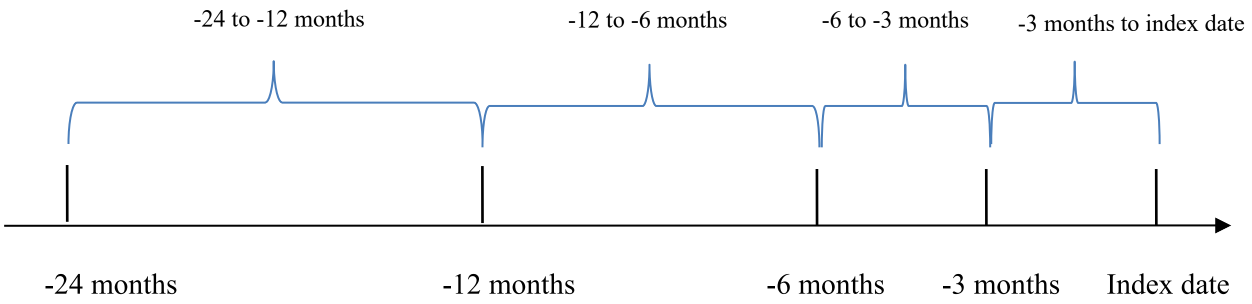


**Figure S1.** Time period of the stress related disease before the diagnosis of IC/BPS (index date).

**Table S1**. ICD-9 diagnostic codes of 33 autoimmune diseases

| Autoimmune Diseases | ICD-9-CM Codes |
|---|---|
| Graves' disease | 242,242.01 |
| Psoriasis | 696, 696.1, 696.8 |
| Systemic lupus erythematosus | 710.0 |
| Rheumatoid arthritis | 714 |
| Ankylosing spondylitis | 720.0 |
| Guillain–Barre´syndrome | 357.0 |
| Sjogren's syndrome | 710.2 |
| Myasthenia gravis | 358.0 |
| Pernicious anemia | 281.0 |
| Hereditary hemolytic anemia | 282 |
| Polyarteritis nodosa | 446 |
| Celiac disease | 579.0 |
| Uveitis | 364.00, 364.01 |
| Polymyalgia rheumatica | 725 |
| Dermatomyositis | 710.3 |
| Hashimoto's thyroiditis | 245.2 |
| Hypersensitivity vasculitis | 446.2, 446.29 |
| Behcet's disease | 136.1 |
| Polymyositis | 710.4 |
| Alopecia areata | 704.01 |

| | |
|---|---|
| Autoimmune hemolytic anemia | 283.0 |
| Multiple sclerosis | 340 |
| Systemic sclerosis | 710.1 |
| Juvenile rheumatoid arthritis | 714.30, 714.33 |
| Goodpasture syndrome | 446.21 |
| Giant cell arteritis | 446.5 |
| Thromboangitis obliterans | 443.1 |
| Arteritis obliterans | 446.7 |
| Kawasaki disease | 446.1 |

**Table S2**. ICD-9 diagnostic codes of sleep disorders

| sleep disorders | ICD-9-CM Codes |
|---|---|
| Alcohol induced sleep disorders | 291.82 |
| Drug induced sleep disorders | 292.85 |
| Nonorganic Sleep Disorder Unspecified | 307.4 |
| Organic sleep disorders | 327 |
| Restless Leg Syndrome | 333.94 |
| Cataplexy and narcolepsy | 347 |
| Unspecified sleep disturbance | 780.5 |
| Lack of adequate sleep | V69.4 |

**Table S3**. The repeat diagnosis of SRDs and odd ratio of the diagnosis of IC/BPS

| | SRD visit at | | | | |
|---|---|---|---|---|---|
| Type of SRD | -24 to -12 months | -12 to -6 months | -6 to -3 months | -3 months to index | Adjusted odds ratio (95% CI) |
| UTI | | | | | |
| | N | N | N | N | Reference |
| | Y (Ever diagnosed from -24 to -3 months) | | | N | 5.33(4.33 to 6.56) |
| | N | N | N | Y | 18.27(13.01 to 25.65) |
| | Y (Ever diagnosed from -24 to -3 months) | | | Y | 50.17(36.95 to 68.13) |
| | N | N | Y | Y | 45.61(19.82 to 104.96) |
| | N | Y | Y | Y | 72.94(28.93 to 183.92) |
| | Y | Y | Y | Y | 101.93(55.52 to 187.14) |
| Peptic ulcer | | | | | |
| | N | N | N | N | Reference |
| | Y (Ever diagnosed from -24 to -3 months) | | | N | 2.70(2.25 to 3.24) |
| | N | N | N | Y | 5.93(3.80 to 9.23) |
| | Y (Ever diagnosed from -24 to -3 months) | | | Y | 3.62(2.72 to 4.83) |
| | N | N | Y | Y | 2.36(1.11 to 5.01) |
| | N | Y | Y | Y | 3.82(1.66 to 8.80) |
| | Y | Y | Y | Y | 3.22(2.06 to 5.06) |
| IBS | | | | | |
| | N | N | N | N | Reference |
| | Y (Ever diagnosed from -24 to -3 months) | | | N | 3.63(2.75 to 4.81) |

| | | | | | |
|---|---|---|---|---|---|
| | N | N | N | Y | 5.44(2.75 to 10.73) |
| | Y (Ever diagnosed from -24 to -3 months) | | | Y | 4.75(2.72 to 8.29) |
| | N | N | Y | Y | 3.49(1.06 to 11.45) |
| | N | Y | Y | Y | 9.56(2.37 to 38.64) |
| | Y | Y | Y | Y | 4.21(1.21 to 14.58) |
| Depression | | | | | |
| | N | N | N | N | Reference |
| | Y (Ever diagnosed from -24 to -3 months) | | | N | 2.21(1.81 to 2.69) |
| | N | N | N | Y | 7.22(4.37 to 11.95) |
| | Y (Ever diagnosed from -24 to -3 months) | | | Y | 3.79(3.00 to 4.79) |
| | N | N | Y | Y | 3.01(1.26 to 7.20) |
| | N | Y | Y | Y | 5.59(2.98 to 10.49) |
| | Y | Y | Y | Y | 3.24(2.38 to 4.40) |
| Sleep disorders | | | | | |
| | N | N | N | N | Reference |
| | Y (Ever diagnosed from -24 to -3 months) | | | N | 2.29(1.91 to 2.73) |
| | N | N | N | Y | 3.57(2.40 to 5.32) |
| | Y (Ever diagnosed from -24 to -3 months) | | | Y | 2.96(2.33 to 3.77) |
| | N | N | Y | Y | 2.44(1.22 to 4.88) |
| | N | Y | Y | Y | 1.67(0.75 to 3.73) |
| | Y | Y | Y | Y | 2.95(2.10 to 4.15) |
| Allergic rhinitis | | | | | |

| | | | | | |
|---|---|---|---|---|---|
| | N | N | N | N | Reference |
| | Y (Ever diagnosed from -24 to -3 months) | | | N | 1.73(1.42 to 2.10) |
| | N | N | N | Y | 2.10(1.23 to 3.57) |
| | Y (Ever diagnosed from -24 to -3 months) | | | Y | 2.08(1.41 to 3.07) |
| | N | N | Y | Y | 5.77(2.00 to 16.67) |
| | N | Y | Y | Y | 1.42(0.52 to 3.88) |
| | Y | Y | Y | Y | 1.50(0.70 to 3.23) |

**Table S4.** morbidities of autoimmune disease among study groups

| Variables | Control | IC/BPS | Univariate OR (95% C.I.) | p |
|---|---|---|---|---|
| n | 4412 | 1103 | | |
| Co-morbidities (within 2 year before index date) of autoimmune disease | 318(7.2%) | 128(11.6%) | 1.69(1.36 to 2.11) | <0.001 |
| Crohn's disease | 60(1.4%) | 28(2.5%) | 1.88(1.20 to 2.96) | 0.006 |
| Sjogren syndrome | 46(1.0%) | 31(2.8%) | 2.72(1.72 to 4.30) | <0.001 |
| Rheumatoid arthritis | 56(1.3%) | 16(1.5%) | 1.15(0.65 to 2.00) | 0.635 |
| Graves' disease | 25(0.6%) | 8(0.7%) | 1.28(0.58 to 2.84) | 0.543 |
| Ankylosing spondylitis | 16(0.4%) | 6(0.5%) | 1.53(0.58 to 4.04) | 0.386 |
| Systemic lupus erythematosus | 9(0.2%) | 6(0.5%) | 2.67(0.95 to 7.49) | 0.063 |
| Hereditary hemolytic anemia | 10(0.2%) | 4(0.4%) | 1.60(0.50 to 5.10) | 0.427 |
| Hashimoto's thyroiditis | 7(0.2%) | 5(0.5%) | 2.86(0.91 to 9.00) | 0.073 |
| Uveitis | 10(0.2%) | 2(0.2%) | 0.80(0.18 to 3.65) | 0.773 |
| Alopecia areata | 8(0.2%) | 2(0.2%) | 1.00(0.21 to 4.71) | 1.000 |
| Polymyalgia rheumatica | 9(0.2%) | 0(0.0%) | - | - |
| Ulcerative colitis | 3(0.1%) | 5(0.5%) | 6.67(1.59 to 27.90) | 0.009 |
| Polyarteritis nodosa | 4(0.1%) | 0(0.0%) | - | - |

The rare autoimmune diseases were not listed, because of the poor statistical power.
